# Factors Affecting the Design of Slow Release Formulations of Herbicides Based on Clay-Surfactant Systems. A Methodological Approach

**DOI:** 10.1371/journal.pone.0059060

**Published:** 2013-03-19

**Authors:** María del Carmen Galán-Jiménez, Yael-Golda Mishael, Shlomo Nir, Esmeralda Morillo, Tomás Undabeytia

**Affiliations:** 1 Agrochemistry and Soil Conservation Department, Spanish Research Council-Institute of Natural Resources and Agrobiology, Seville, Spain; 2 The Seagram Center for Soil and Water Sciences, The Robert H. Smith Faculty of Agriculture, Food and Environment, Hebrew University of Jerusalem, Rehovot, Israel; University of Crete, Greece

## Abstract

A search for clay-surfactant based formulations with high percentage of the active ingredient, which can yield slow release of active molecules is described. The active ingredients were the herbicides metribuzin (MZ), mesotrione (MS) and flurtamone (FL), whose solubilities were examined in the presence of four commercial surfactants; (i) neutral: two berols (B048, B266) and an alkylpolyglucoside (AG6202); (ii) cationic: an ethoxylated amine (ET/15). Significant percent of active ingredient (*a.i.*) in the clay/surfactant/herbicide formulations could be achieved only when most of the surfactant was added as micelles. MZ and FL were well solubilized by berols, whereas MS by ET/15. Sorption of surfactants on the clay mineral sepiolite occurred mostly by sorption of micelles, and the loadings exceeded the CEC. Higher loadings were determined for B266 and ET/15. The sorption of surfactants was modeled by using the Langmuir-Scatchard equation which permitted the determination of binding coefficients that could be used for further predictions of the sorbed amounts of surfactants under a wide range of clay/surfactant ratios. A possibility was tested of designing clay-surfactant based formulations of certain herbicides by assuming the same ratio between herbicides and surfactants in the formulations as for herbicides incorporated in micelles in solution. Calculations indicated that satisfactory FL formulations could not be synthesized. The experimental fractions of herbicides in the formulations were in agreement with the predicted ones for MS and MZ. The validity of this approach was confirmed in *in vitro* release tests that showed a slowing down of the release of *a.i*. from the designed formulations relative to the technical products. Soil dissipation studies with MS formulations also showed improved bioactivity of the clay-surfactant formulation relative to the commercial one. This methodological approach can be extended to other clay-surfactant systems for encapsulation and slow release of target molecules of interest.

## Introduction

Clay minerals are widely used in numerous industrial applications as fillers in production of paper, plastics; heat insulators in building industry; nacreous pigments in cosmetics; corrosion proof agents in coatings; anti-caking agents in chemical industry; UV-, heat-stable and under-water agents in paintings, etc. [1]

Recently there is a growing use of clays in delivery technology in order to confer slow release properties of the active ingredient, which is of particular interest in pharmaceutical and agrochemical industry. Levis and Deasy [2] showed prolonged release of the antihyperthensive propranolol from loaded halloysite tubes. Park et al. [3] intercalated donepezil into several layered clays and the formulated products showed a biphasic release pattern with an initial burst effect followed by a slow release of the drug. Clay-polymer complexes were also designed for slowing down the release pattern of the drug docetaxel [4]. The polymer improved the mechanical and rheological properties of the formulations [5].

Several clay-based approaches for slow release have been used for pesticides, e.g., modification of the clay surface from hydrophilic to hydrophobic by preadsorbing organic cations and, therefore, enhancing the affinity for hydropobic herbicides [6]–[8]. Other strategies involve trapping of the herbicide by coagulation of delaminated clay particles [9]; sorption on thermally treated-clay [10]; intercalation of copolymers of the pesticide [11]; encapsulation into a polymer-clay [12] and clay-gel [13] matrix, etc.

In general, clay minerals are incorporated in crop protection products as rheological modifiers to give large stability over the productś lifespan, thus avoiding syneresis. It has been previously reported that incorporation of active ingredients in the micelles and vesicles formed by surfactant molecules which adsorbed on clays, such as montmorillonite, provided a slow release system [14]–[16]. The fraction of the pesticide encapsulated in surfactant-clay complexes prolonged their action. Clearly the encapsulated fraction and the released pattern depend on the chemical properties of both the surfactant and the pesticide, of the type of clay mineral and of the range of concentrations used.

In the current work, we test the applicability of a methodological approach for the design of slow release formulations based on clay-surfactant. The following elements were considered for formulation optimization: (i) determination of the solubility of the active ingredient in surfactant systems; (ii) determination of the binding affinity of the surfactants to the clay surface; (iii) optimization of surfactant sorption at several clay/surfactant ratios; and (iv) estimating the amount of active ingredient sorbed for that clay/surfactant ratio. We tested the approximation that the same proportionality between the active ingredient included in micelles and the surfactant in solution, could be retained in the presence of clay. The applicability of this approach was examined for three herbicides (MS, MZ and FL) and four surfactants employed in commercial formulations (neutral: an alkypolyglucoside, two ethoxylated alcohols and one cationic ethoxylated amine). The clay mineral used was sepiolite, which can form weak gels at very low concentrations yielding good viscosity properties [17].

## Materials and Methods

### Materials

Metribuzin [4-amino-6-ter-butyl-3-methylthio-1,2,4-triazin-5(4H)-one] (MZ), mesotrione [2-(4-mesyl-2-nitrobenzoyl)cyclohexane-1,3-dione] (MS) and flurtamone [(2RS)-5-methylamino-2-phenyl-4-(α,α,α-trifluoro-m-tolyl)furan-3(2H)-one] (FL) were purchased from Sigma-Aldrich Co., St Louis, MO). The commercial formulation of MS (Callisto, 10% w/V) was a gift from Syngenta Spain. The surfactants AG6202, Ethomeen T/15 (ET/15), Berol 266 (B266) and Berol 048 (B048) were kindly provided by Akzo Nobel Co. (Amsterdam, The Netherlands). HPLC grade-methanol was purchased from Sigma-Aldrich (Sigma Chemical Co., St Louis, MO); H_3_PO_4_ (85% w:v) and HPLC grade-acetonitrile was obtained from Teknokroma S.A. (Barcelona, Spain). Sepiolite (Pangel S9) was obtained from Tolsa S.A. (Madrid, Spain). Sepiolite is a hydrated Mg phylosilicate with fibrous morphology formed by blocks and cavities (tunnels) growing in the direction of the fibers [18]. Each structural block is composed of a central Mg sheet sandwiched between two tetrahedral silica sheets. Silanol groups are covering the external surface which is accessible to reagents and acting as neutral sorption sites. In addition, certain isomorphic substitutions in the tetrahedral sheets are responsible for the cationic exchange capacity of this clay (0.15 cmol_c_ kg^−1^).

**Figure 1:**
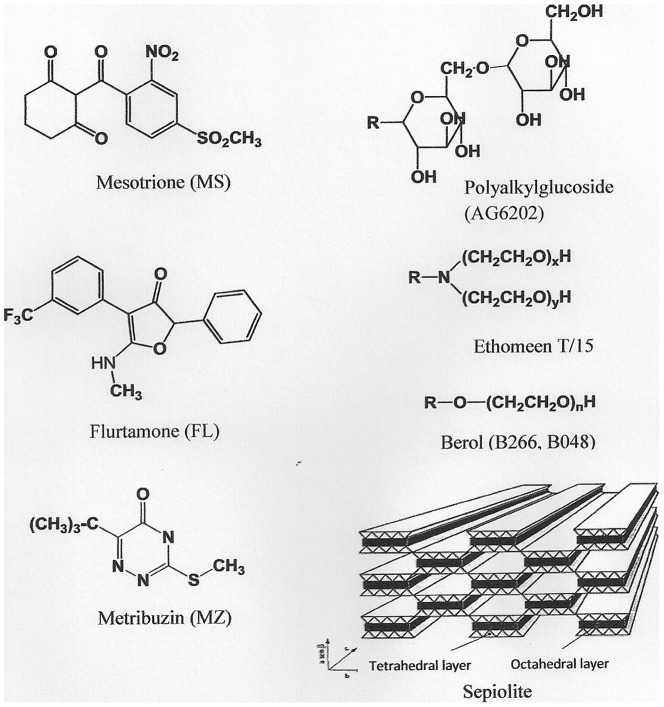
Sepiolite structure and structural formulas of the herbicides and surfactants.

Figure 1 shows the sepiolite structure and the structural formulas of the herbicides and surfactants. Tables 1 and 2 list properties of the surfactants and herbicides, respectively.

**Table 1:** Surface and chemical properties of the surfactants.

	B048	B266	ET/15	AG6202
M.W.	640	400	480	370
R	C_13_	C_10_	C_17_	C_8_
n. ethoxyl units	10	5.5	5	–
HLB§	13.5	12.1	9.2	11.2
cmc (mM)§	0.16	0.075	0.041	37.83
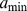 (nm^2^)^f^	0.64	0.50	0.19	0.20

§ HLB, hydrophilic-lipophilic balance; cmc, critical micellar concentration.

f Determined from surface tension measurements.

**Table 2:** Physico-chemical and environmental properties of the herbicides.^a^

	FL	MZ	MS
M.W.	333.30	214.29	339.32
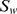 (at 20°C) (mg L^−1^)	10.7	1165	160
Log Kow^b^	3.2	1.65	0.11
pKa	–	0.99	3.12
Freundlich parameters			
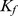	0.66–7.19	0.018–1.9	0.33–4.48
1/n	0.9	0.89–1.52	–
DT_50_ (days)	48–211	5.3–17.7	32
GUS leaching index	2.59	2.57	3.43
Mobility	Transition state	Transition state	High leachability

a. From the IUPAC-Pesticide properties data base (http://sitem.herts.ac.uk/aeru/iupac/).

b. Kow, octanol-water partition coefficient at pH 7 and 20°C.

The upper horizon of a clay soil from Lebrija (Sevilla, Spain) with a 1.1% organic matter content and a basic pH (7.6) was collected and passed through a 2 mm sieve before use.

### Surface tension

Surface tension (*γ*) measurements were performed with a LAUDA TD 3 tensiometer using Du Nuoy ring detachment method. The ring was cleaned with ethanol and flamed after every measurement. For each measurement at least five readings were taken and the mean γ value was recorded. Before each experiment the instrument was calibrated and checked by measuring the surface tension of distilled water. Surface tension measurements of ET/15 solutions were performed in the presence of 0.01 M NaCl as a background electrolyte. The minimum area occupied by a surfactant molecule at the air/solution interface, 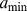, was estimated from its maximum surface excess concentration (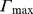) as determined from the Gibb´s adsorption equation.

### Solubility studies

10 mL of surfactant solutions at concentrations ranging up to 20 g/L were added in duplicate to a suspension of each herbicide beyond its solubility limit, and the suspensions were shaken for 1 week at 25°C. Then the suspensions were let to settle down; the supernatant was removed and filtered through 0.20 µm PTFE membranes, and the herbicide analyzed. The solubility curves were constructed by plotting the amount of herbicide solubilized versus the amount of surfactant employed.

A solubility enhancement factor of the herbicides by the use of surfactants was determined from 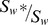, where 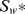 is the apparent solubility at a 20 g/L surfactant concentration and 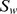 is the intrinsic herbicide solubility in water. 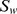
is 0.41 mM for MS, 5.13 mM for MZ and 0.02 mM for FL.

### Sorption studies

The adsorption of surfactants on the clay was carried out in glass vials in duplicate by mixing 15 ml of surfactant solutions whose concentrations were up to 12 g/l with 24 mg of sepiolite. The clay concentration in the vials was 1.6 g/L. After shaking for 24 h at 20°C, the suspensions were centrifuged at 12000 g for 10 min and the supernatants removed. Kinetic experiments showed that equilibrium was reached within 24 h shaking of clay-surfactant suspensions. Preliminary experiments with only surfactant solutions were performed to determine the operating conditions for centrifugation by avoiding sedimentation of the surfactant molecules. The pellets were dry-frozen and surfactant concentration in the pellets was determined by elemental C analysis.

Surfactant sorption was modeled by using the Langmuir-Scatchard equation:
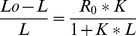



Where 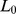
and 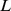 denote the molar concentration of total and free surfactant, respectively, 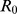
is the molar concentration of sorption sites and 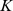 is the binding coefficient.

Sorption of the herbicides was analogously performed on sepiolite. Clay concentration was again 1.6 g/L. The added herbicide concentrations were up to 20 mg L^−1^ for MZ and MS, and up to 5 mg L^−1^ for FL.

Sorption-desorption isotherms were fitted to the logarithmic form of the Freundlich equation:
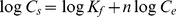
were 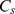 (mg g^−1^) is the amount of adsorbed herbicide, 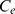 (mg mL^−1^) is the equilibrium concentration of herbicide, and 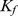 and *n* are constants that characterize the relative sorption capacity and sorption intensity, respectively.

### Zeta potential measurements

The surfactant-clay composites were redispersed at a concentration of 1.6 g/l. The samples were allowed to equilibrate for 1 h and few milliliters of dispersion were measured. The temperature of the samples was 25±1°C, ξ was measured using a Zetasizer Nanosystem (Malvern Instruments, Southborough, MA). The ξ value was deduced from the mobility of the particles using the Smoluchowski equation.

### Preparation of herbicide formulations

Herbicide-clay-surfactant formulations were prepared by dissolving several amounts of the herbicides in surfactant solutions, which were later added to sepiolite. The concentrations of herbicides and surfactant were determined from solubility diagrams and the corresponding surfactant sorption on sepiolite. Optimization of herbicide in the formulation considered both the enhanced solubility of the herbicide and sorption of the complexes formed in solution on the clay surface. The employed concentrations are listed in Table 3. The calculations of *a.i*. (w/w%) in Table 3 were as follows. As an approximation to the amount of herbicides in micelles of the surfactant, the solution concentrations of herbicides in the absence of surfactants were subtracted from their enhanced amounts in the presence of surfactants. The adsorbed amounts of surfactants were calculated by means of the Langmuir equation. The amount of a herbicide in the formulations surfactant/herbicide/clay was calculated by multiplying its amount in the micelles by the fraction of adsorbed micelles. After shaking for 24 h the suspensions were centrifuged at 12000 g for 10 min, and the pellets were dry-frozen yielding the herbicide formulations. 10 mg of these formulations were extracted with 20 mL of methanol to determine their active ingredient (*a.i*.) content. A nomenclature for herbicide-clay-surfactant formulations was introduced (see Table 3) where the first two letters indicate the herbicide (MS: mesotrione; MZ: metribuzin) followed by letters indicating the surfactant used, the first number denotes the surfactant concentration, the second one gives the initial herbicide concentration and the third one the clay concentration used.

**Table 3:** Clay-surfactant formulations.

Herbicide	Surfactant, conc. (g/L)	Herbicide added (mM)	Clay conc. (g/L)	Notation of formulation	Calculated *a. i*. (w/w%)	Experimental *a. i*. (w/w%)
Mesotrione	Berol 048, 0.07	0.69	0.5	MSB048 0.07/0.69/0.5	3.9	6.0±0.4
	Berol 266, 1	0.48	1	MSB266 1/0.48/1	1.9	2.7±0.2
	ET/15, 12	23.00	5	MSET 12/23/5	19.6	15.8±0.8
	ET/15, 12	23.00	15	MSET 12/23/15	18.8	12.5±0.4
	ET/15, 12	23.00	20	MSET 12/23/20	17.7	11.1±0.3
Metribuzin	Berol 048, 3	6.44	1.6	MZB048 3/6.44/1.6	2.4	2.8±0.1
	Berol 266, 5	7.55	1.6	MZB266 5/7.55/1.6	3.2	4.6±0.1
	ET/15, 3	6.29	3	MZET 3/6.29/3	2.3	3.9±0.1
	ET/15, 5	6.95	5	MZET 5/6.95/5	3.8	3.2±0.1
	ET15/20	10	20	MZET 20/10/20	1.8	4.5±0.1

### Studies of release in water

Release tests of technical grade herbicide and selected clay-based formulations were performed in duplicate with a rotating paddle apparatus (Sotax). For each formulation 5 mg of herbicide were added to 1 L of deionized water at 25°C and stirred at 50 rpm. At appropriate time intervals from 0 to 96 h, samples were taken, immediately passed through PTFE filters and the herbicide was analyzed.

### Soil dissipation

MS dissipation was carried out in pot experiments with the commercial formulation and one of the clay-surfactant-herbicide formulations (MSET/15 12/23/5). Seeds of sunflower (*Helianthus annuus*) were cultivated under hydroponic conditions. Then, 10 seedlings were transplanted after appearing the first true leaf to pots of 10 cm diameter filled with 0.32 kg of the clay soil. The formulations were sprayed on the soil at the recommended dose (100 g ha^−1^), and at ½- and 2-fold of that. After 10 days in a climatic chamber, the bleaching intensity was obtained by measuring the chlorophyll content and determining the inhibition percentage in relation to controls (no herbicide was added). The chlorophyll content was determined by cutting the fresh shoot of plants from the starting of the first true leaf and extracted with 12 mL of *N,N*-dimethylformamide. The solutions were incubated for 48 h, and the chlorophyll (*a* and *b*) content was measured by vis spectroscopy at 664 and 647 nm and related to the weight of the extracted amount. The experiments were done in triplicate for each formulation and dose.

### Herbicide analysis

Herbicides were analyzed by HPLC (Shimadzu Model 10A) equipped with a PDA detector. The reverse phase column was a 15 cm Kromasil 100 C18. The flow rate was 1.0 mL min^−1^. The mobile phase was 40% acetonitrile and 60% water containing 0.1% H_3_PO_4_. The wavelengths were set at 220 nm for FL, 254 nm for MS and 230 nm for MZ. The retention times were 15.19, 3.05 and 2.86 min for FL, MS and MZ, respectively.

## Results and Discussion

### Herbicide solubility

**Figure 2:**
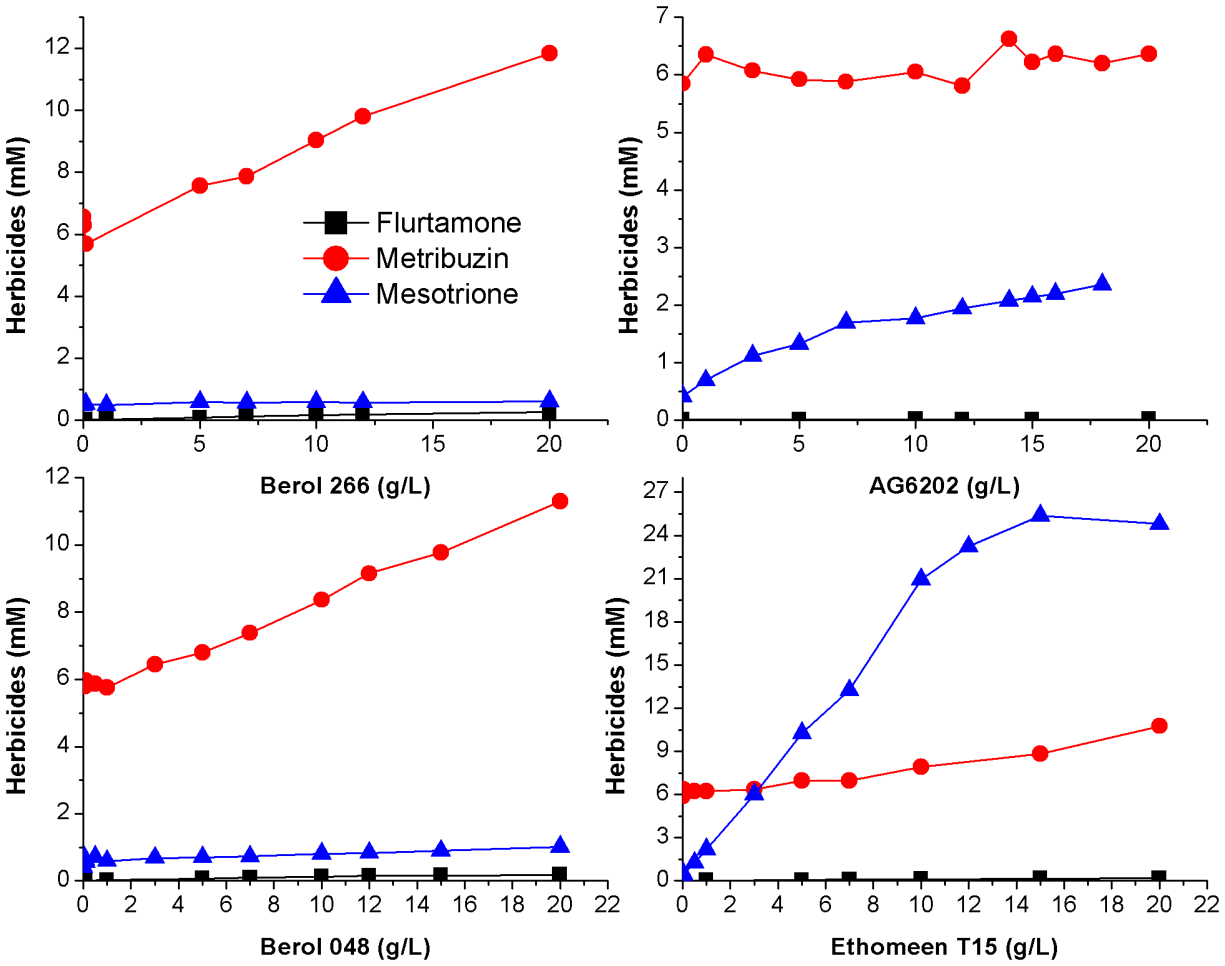
Phase solubility diagrams of the herbicides in the presence of surfactants.

Solubility enhancement of the herbicides (Table 4, Figure 2) was strongly influenced by their polarity and chemical structure as well as by their compatibility with the surfactant molecules (Figure 1). The data in Table 4 showed that MS and FL were appreciably solubilized by ET/15, whereas in the case of berols (B048, B266), the largest solubility enhancement factors were obtained for FL. In contrast, the solubility factors of MZ were low in all surfactants used and ranged from 1.09 to 2.02.

**Table 4:** Solubility enhancement factor for the herbicide-surfactant systems.

	MS	MZ	FL
B048	2.40	1.93	18.9
B266	1.29	2.02	22.5
ET/15	59.0	1.84	18.1
AG6202	5.90	1.09	1.15

B266 solubilized the more hydrophobic herbicide FL relative to B048 and ET/15, which did not follow the HLB values of the surfactants (Table 1) and revealing other factors are influencing the solubilizing capacity of the surfactants. Probably, interactions of the herbicide molecules with the polar groups of the surfactants play an important role. Ethoxyl units are strongly hydrated and may form water bridges with polar moieties of the herbicide molecules, resulting in an enhanced solubilization. This mechanism is of less importance for very hydrophobic herbicides. Therefore, the enlarged solubilization of FL by B266 was due to its lower number of ethoxyl units, facilitating FL penetration and interaction with the micellar core.

When the phase solubility diagrams of the herbicides in the presence of surfactants were examined (Figure 2), MZ solubility increased with increasing concentration of the two berols from 0 to 20 g/L, the total amount dissolved was respectively increased from 6 up to 12 mM. MS solubility was greatly enhanced in the presence of ET/15 from 1 to 25 mM when increasing concentration of ET/15 from 0 to 20 g/L. This large solubilization was mainly driven by electrostatic interactions. MS is a weak acid (pKa = 3.12) remaining mostly as anionic species at the equilibrium pH (5.4) in the solubility studies, whereas the surfactant molecules of ET/15 were positively charged (pKa = 8.5). Finally, phase solubility of FL was almot not affected by any surfactant, the total amount dissolved was very small. These values seem to be contradictory to those in Table 4. For example, the solubility enhancement factors for FL with berols were larger than those for MZ. However, the solubility enhancement factors were calculated relative to the intrinsic solubility of the herbicides. FL has a very small solubility (10.7 mg/L), therefore the total amount of herbicide in solution dissolved for a fixed concentration of berols was always smaller than with MZ, which has a high value of water solubility (1165 mg/L). In the design of clay-based formulations, it is desirable to have (i) a large ratio between the amount of herbicide dissolved and surfactant used, (ii) a high probability of sorption of the herbicide-surfactant complexes to the clay and, (iii) a large amount of the active ingredient (*a.i*.) in the formulations. Consequently, the amount of MZ in solution dissolved for the same amount of surfactant will be much larger than with FL, and also the probability of sorption of the surfactant-herbicide complexes, yielding formulations with larger *a.i.*

In general, enhanced solubility of the herbicides by the surfactants occurred through incorporation of the herbicide molecules into the micelles formed in solution, as no solubilization was noticed for surfactant concentrations below their cmc, with the exception of AG6202, which showed a monotonic increase in the solubilized amount of mesotrione at concentrations below its high cmc (14 g/L). This enhanced MS solubilization may be due to the formation of large premicellar aggregates that have been often observed [19], [20], with which the herbicide would be interacting.

### Surfactant sorption

**Figure 3:**
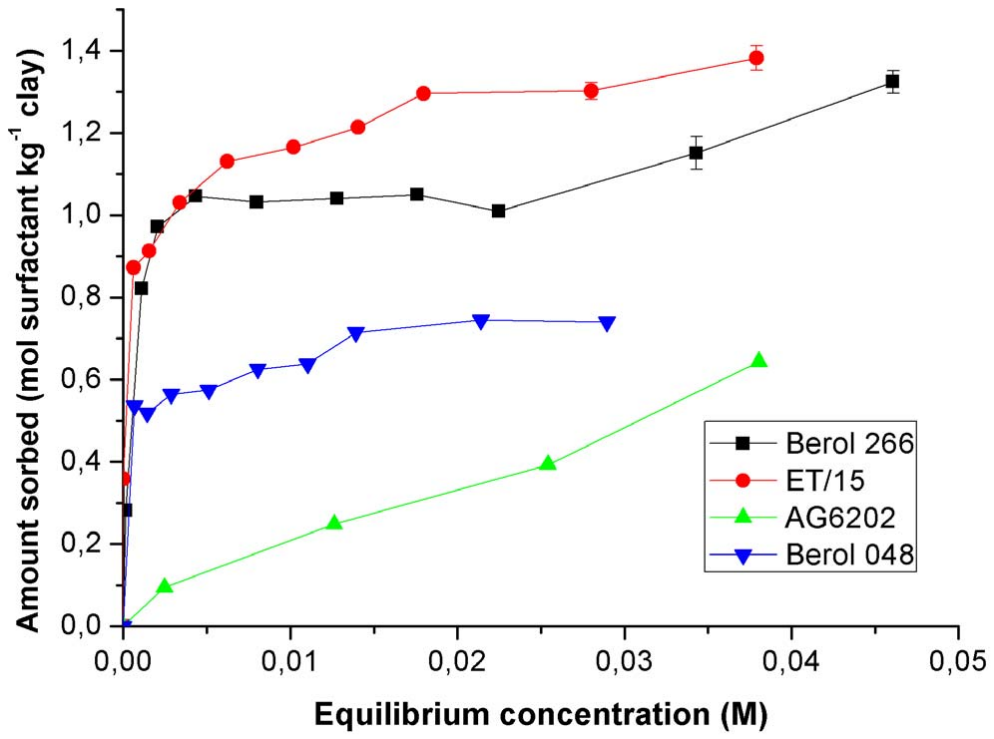
Sorption isotherms of surfactants on sepiolite.

The sorption capacity of surfactants by the clay mineral was affected by the type of surfactant (Figure 3). A larger sorption was observed with ET/15, followed by berol surfactants, the lowest adsorption was obtained for polyalkylglucoside AG6202. Higher loading of the surfactant AG6202 on sepiolite was not possible by increasing the total amount since its adsorption was limited due to precipitation during centrifuging at higher concentrations. Consequently, this surfactant was discarded for preparation of herbicide formulations because of its low sorption on the clay and its generally poor efficacy for enhanced solubility of herbicides. The sorption of the surfactants on the sepiolite was well-fitted to Freunlich equation with the exception of B266 (Table 5). The comparison between 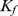 values was only allowed for B048 and ET/15 due to its similar *n* values, indicating a higher loading and thus affinity for ET/15 on sepiolite over B048.

**Table 5:** Parameters of the Freundlich equation describing the adsoption isotherms of the herbicides and surfactants on sepiolite.

	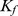 (mg^1−(1/n)^ mL^1/n^ g^−1^)	1/n	R^2^
MS	43.65	0.75	0.95
FL	6.33	0.49	0.98
MZ	4.73	1.12	0.97
B048	346.73	0.10	0.86
B266	295.12	0.20	0.74
ET/15	467.33	0.12	0.99
AG6202	34.67	0.67	0.98

**Figure 4:**
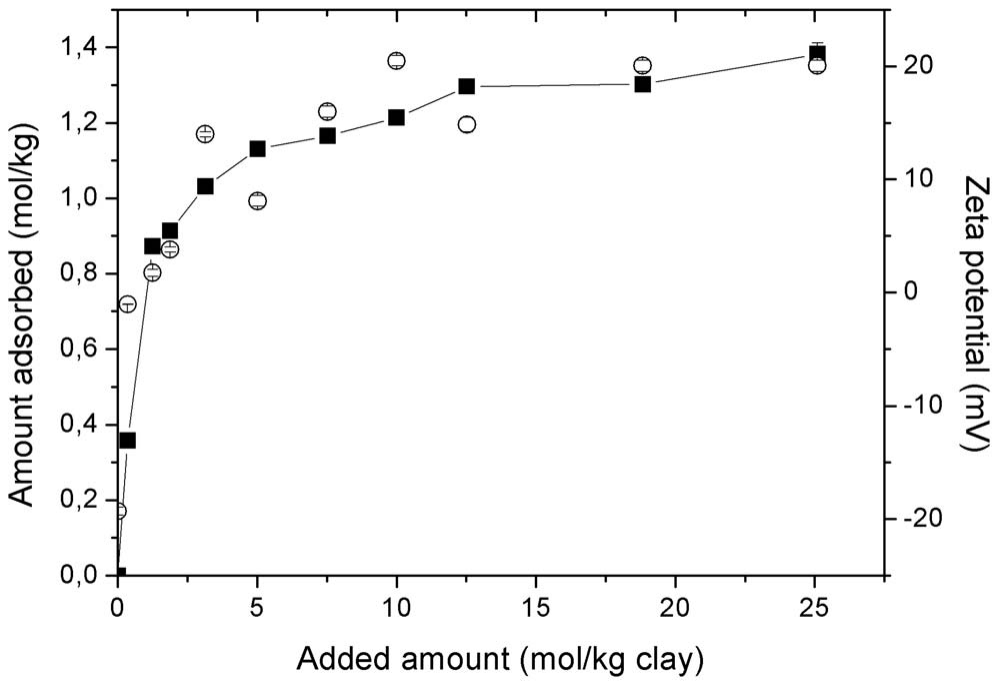
Zeta potential (ζ) of clay-ET/15 complexes as a function of ET/15 sorption on sepiolite. Open and closed symbols denote the zeta potential and sorbed amount, respectively.

ET/15 molecules were protonated at equilibrium pH, so that they were first sorbed on sepiolite by ion exchange with Mg as reported for quaternary and primary amines. For higher loads adsorption was followed by combination of this electrostatic mechanism with chain-chain interactions [21], [22]. Li et al [23] showed formation of admicelles in the sorption of hexadecyltrimethylammonium on the external surface of fibrous clay minerals, resulting in a positively charged surface. Zeta potential was examined for the ET/15-sepiolite complexes formed during ET/15 sorption onto sepiolite (Figure 4). Charge reversal was observed for added concentrations of 1.25 mol/kg; the zeta potential was close to the p.z.c. and slightly positive, and the sorbed amount of ET/15 was 1.2-fold larger than the CEC of the sepiolite. These results indicate an initial sorption of the surfactant by cationic exchange and then, the sorbed monomers of the surfactant serve as nuclei for the development of surface aggregates (admicelles), in an arrangement where a large fraction of the positively charged headgroups is not located at external surfaces.

In the case of non-ionic surfactants, their sorption on clay minerals has been reported to occur through hydrogen bonding with the oxygen atoms of the silicate surface, bonding to the interlayer cations through a water bridge, direct coordination or ion-dipole interaction with the exchangeable cations, and Van der Waals interactions between the alkyl chain of the surfactant and clay surface areas with low charge density [24]–[26]. Shen [27] observed a high sorption of an alkylphenol exthoxylated surfactant on silicon oxide over aluminium and iron oxides, which was due to hydrogen bonding with the strongest Bronsted acid sites of SiO_2_. Because of the fibrous structure of sepiolite, sorption of the non-ionic surfactants would be expected to occur through H-bonding to silanol groups covering the surface, similarly to sorption on silicon oxide.

In Figure 3, sorption of B048 on sepiolite reached 0.74 mol kg^−1^ which was close to the amount of neutral sites in sepiolite, 0.6 mol kg^−1^ as determined from grafting reaction of organoclorosilanes on the silanol groups [28]. This value is somewhat larger than that of 0.51 mol kg^−1^ estimated from the analysis of the sorption of Triton X100 on sepiolite by Rytwo et al. [29]. These authors pointed out that geometrical considerations marginally allowed the entry of Triton X100 molecules into the channels of the sepiolite, and there was no cooperativity in the binding of the non-ionic molecules. Albeit B048 poses the same number of ethoxyl units as Triton X100, its larger hydrophobic moiety will modify its sorption pattern as compared to Triton X100. It has been reported that sorption of non-ionic surfactants on hydrophilic surfaces will result in a monolayer from monomer sorption on the surface. In the case of a relatively strong interaction between the surfactant hydrophilic moieties and those of the surface as in silica or oxides, the monomers will adopt a conformation with the headgroup anchored to the surface and the alkyl chain displaced from the surface and protruded into the solution. An increase in the sorption will cause a vertical orientation of the alkyl chains with the further development of aggregate structures (hemimicelles) by interaction between surfactant molecules [30], [31]. A calculation of the packing area per molecule of B048 at the sorption plateau yielded 0.60 nm^2^, which is quite close to 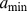
(Table 1). indicating the development of a monolayer with the molecules standing up with no further aggregation process.

With B266 (Figure 3), a plateau is observed at 1.04 mol kg^−1^, and the calculation of the packing area in the plateau yielded a similar value (0.45 nm^2^) to 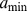. However, unlike B048, a further increase in the sorption after the plateau is noticed, revealing the formation of surfactant aggregates on the sepiolite surface. Formation of surface aggregates due to lateral interactions between surfactant molecules on the sepiolite surface is energetically more favoured for B266 than for B048, as reflected by its larger hydrophobicity (lower HLB value). Correspondingly, sorption of B266 on sepiolite was larger than that of B048, despite its lower content of ethoxyl groups, and thus, its reduced capability to form H-bonding to surface groups of sepiolite. A more dense packing enhances the energy of interaction between the alkyl chains, which apparently exceeds the interaction energy between the surfactant molecules and the clay surface.

**Figure 5:**
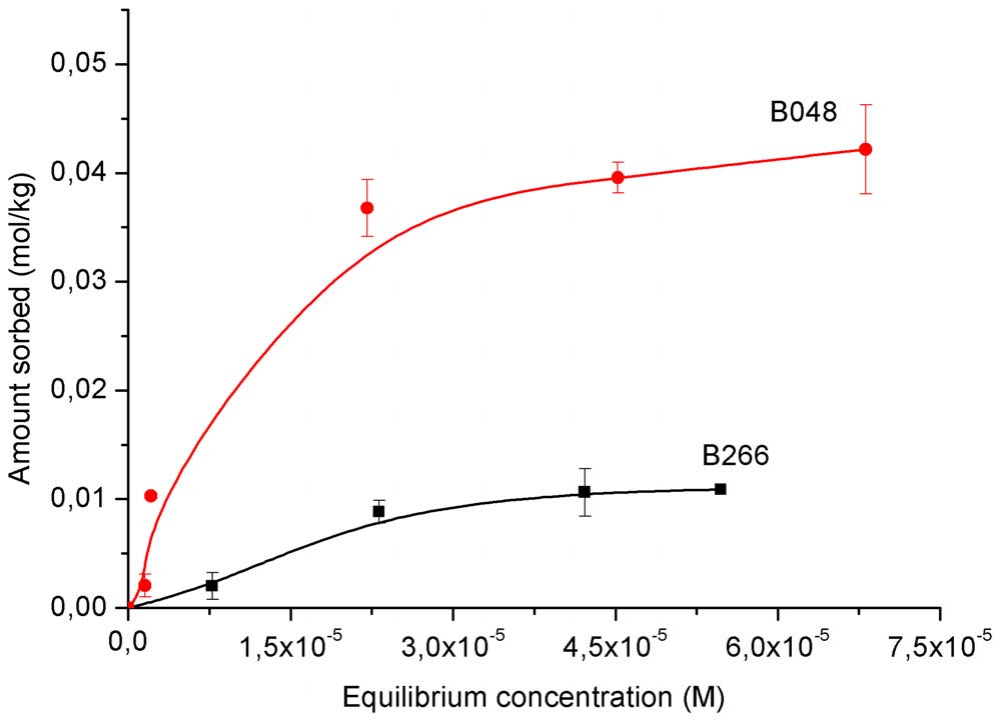
Sorption isotherms of surfactants on sepiolite at added concentrations below their cmc.

In order to elucidate the mechanisms involved in the sorption of the surfactants on the clay, their sorption was also performed at added concentrations of the surfactant below the cmc for the same clay concentration (1.6 g L^−1^); the amount of surfactant in solution was determined by surface tension measurements. Whereas in the case of ET/15, all the molecules were sorbed on the clay, exhibiting a H-type isotherm (data not shown), an adsorption plateau was observed both for B266 and B048 (Figure 5), which was several-fold lower than their higher sorption values recorded previously from sorption as micelles (Figure 3). This indicated that the sorption of non-ionic surfactants at relevant concentrations as in Figure 3, was mainly occurring as micelles, which decomposed after interaction with the clay surface, yielding a monolayer of sorbed surfactant. These results were in contrast to those from previous studies by Levitz [32] and Cases and Villieras [33], who reported that the adsorption layer was built from isolated molecules of the surfactant arriving from aqueous solution and not by direct adsorption of micelles. Surfactant sorption as micelles was also observed for the sorption of cationic surfactants on the negatively charged clay mineral montmorillonite [34], [35]. These authors showed that at high surfactant/clay ratios micelles were adsorbed without undergoing premature decomposition, whereas in the presence of an excess of clay particles over the surfactant concentration, the rapid sorption of monomers on the clay particles resulted in decomposition of the micelles. Likewise, surfactant loading from monomer sorption was lower than sorption as micelles. The effect of the surfactant/clay ratio in the sorption of surfactants on clays is a critical factor.

### Preparation of slow release herbicide formulations

The design of slow release herbicide formulations requires an optimization of two processes: (i) increased solubilization of the herbicide in surfactant solutions; and (ii) high sorption on the clay of the surfactants encapsulating the herbicide molecules. The sorption of surfactants was modeled by using the Langmuir-Scatchard equation. This provided a binding coefficient for each surfactant, that allowed to predict their sorption for several surfactant/clay ratios. The validity of this approach has been extensively proven for other surfactants [16], [36]. In a second stage, once the amount of sorbing surfactant was determined for particular surfactant/clay ratios, the maximal amount of herbicide which could be sorbed for the employed surfactant concentration was calculated from the solubility studies by considering the same ratio between the solubilized (included in micelles) herbicide and the surfactant in solution as in the formulation with the clay. This assumption guided the choice of herbicide/clay/surfactant concentrations to be used for the preparation of the formulations.

As in Nir et al. [37], only one fitting parameter was used in the modeling of the adsorption isotherms by using the Langmuir-Scatchard equation, the binding coefficient 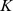
In the determination of the binding coefficients, the concentration of adsorption sites of sepiolite (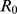) was fixed for each surfactant to be the largest sorbed amount from Fig. 3. The binding coefficients were 900 M^−1^ for Berol 048, 680 M^−1^ for Berol 266 and 950 M^−1^ for ET/15. The fit was reasonably good, yielding low values of the root mean square error (RMSE), 0.027 mol kg^−1^ for Berol 048, 0.084 mol kg^−1^ for Berol 266 and 0.063 mol kg^−1^ for ET/15.

Table 3 shows an estimation of the *a.i*. content by employing several herbicide/clay/surfactant ratios, which were calculated from the binding coefficients as well as from the solubility diagrams. Formulations were prepared for MS and MZ, but not for FL, since in this case the calculations yielded formulations with very low *a.i*. content (<1% w:w). The low herbicide content of FL formulations arises from its very low dissolved amount versus the amount of surfactant used.

**Figure 6:**
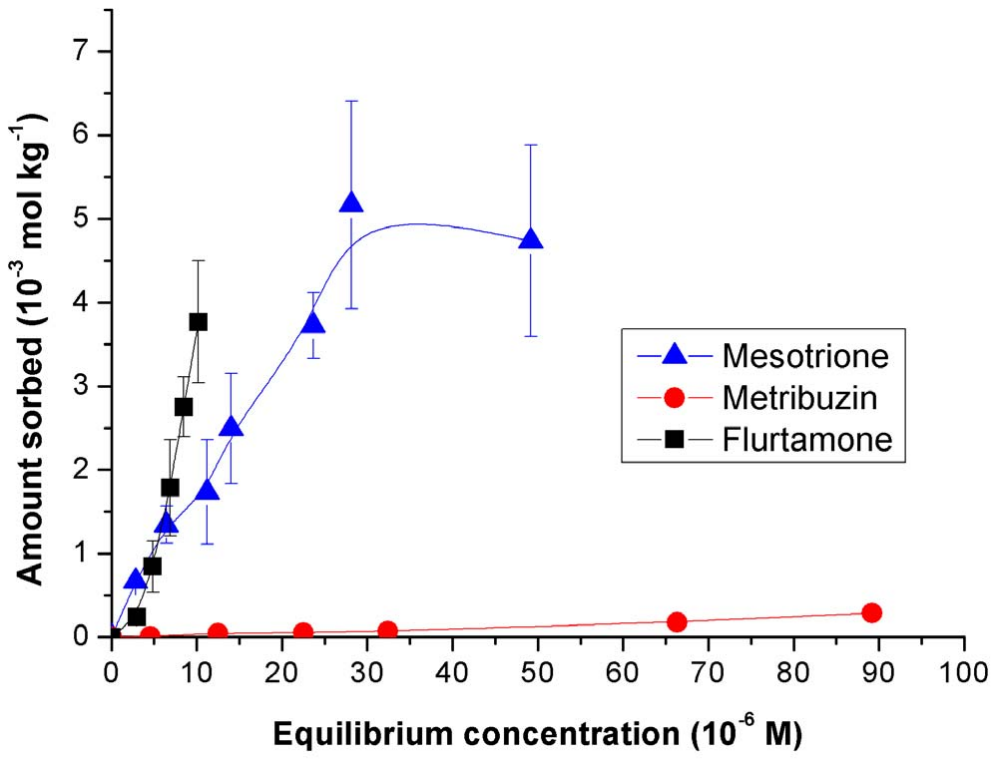
Sorption isotherms of the herbicides on clay.

The herbicides' affinity on the clay mineral was very little (Figure 6). The sorption isotherms of the herbicides were also fitted to Freundlich equation (Table 5), and the fitting was pretty good (R^2^∼0.95–0.98). Again, with the exception of MZ, no comparison in 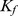 values may be established with reported values because of the large differences in *n* values. MZ was classified as a mobile herbicide with 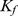 values ranging from 0.018–1.9 mL g^−1^ (Table 2). The experimental 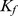 value (4.73 mL g^−1^) on sepiolite was higher but still indicative of poor retention. This value was quite similar for those found in topsoils presenting small sorption, great risk of leaching and fast degradation of MZ: 3.17–3.26 mL g^−1^ [38], 1.49–3.33 mL g^−1^ [39].

Consequently, sorption of the herbicides on the clay occurred mostly through their inclusion in micelles. Overall, the predicted amounts of MS and MZ corroborated reasonably well with the experimental ones (Table 3). The formulations of MS based on ET/15 reached high *a.i.* contents, as much as 15%, whereas smaller contents were obtained for its formulations based on berols or for MZ formulations.

The experimental *a.i.* content of MZ for the clay-surfactant formulations was several-fold lower compared to MS. The lower *a.i*. levels which could be achieved with MZ formulations are due to its relatively large solubility, which implies that a relatively smaller fraction could be included in micelles. Furthermore, table 4 shows that unlike MS, solubility enhancement of MZ was not large in the presence of the surfactant ET/15.

Overall, MS formulations can be designed for several surfactants at optimal ratios between the concentrations of herbicide, surfactant and clay, thus yielding complexes with high *a.i*. contents that are relevant for agricultural applications. In contrast, clay-based formulations of MZ yielded very low *a.i*. contents for all the surfactants selected in the current study.

### Herbicide release in water

**Figure 7:**
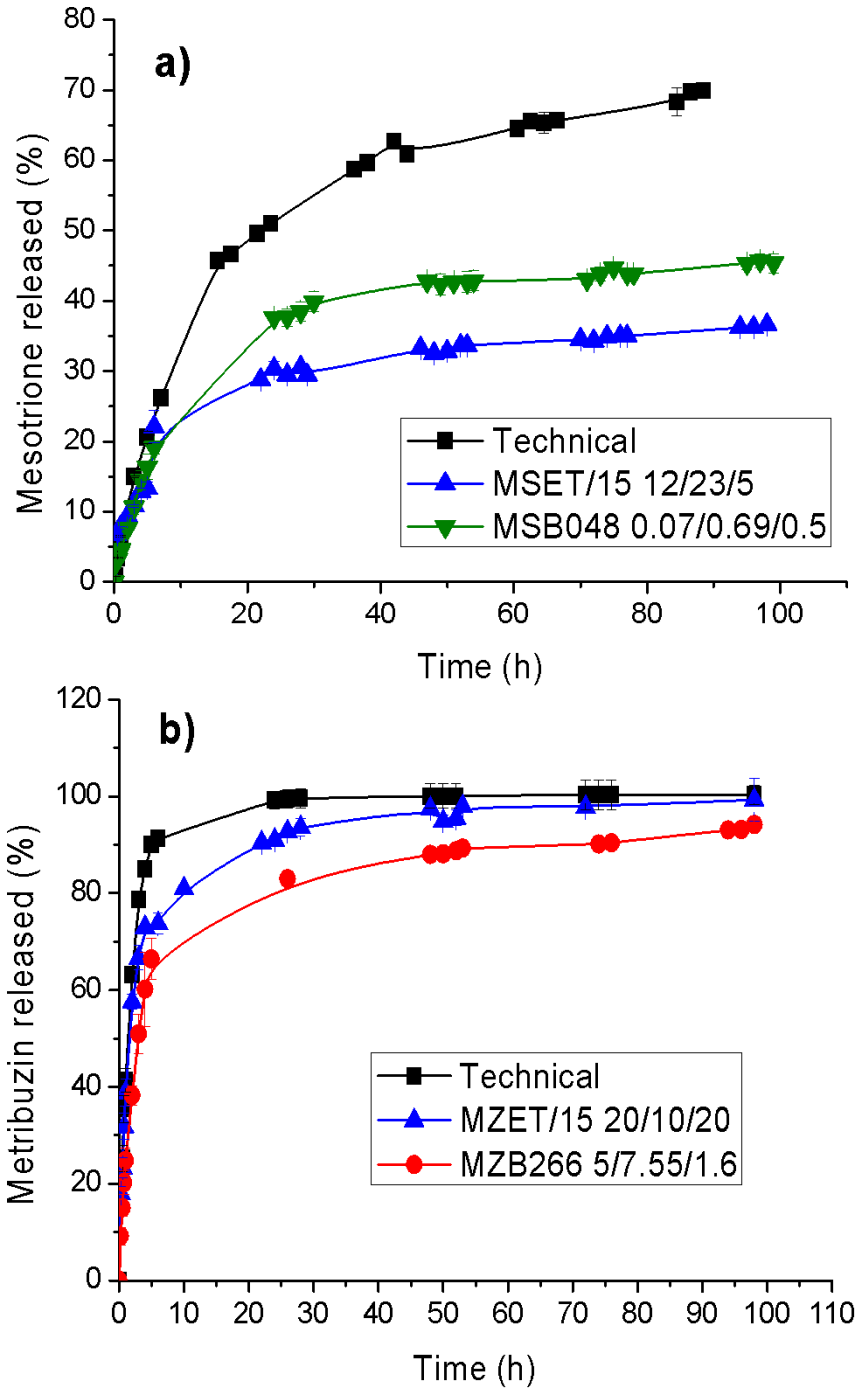
*In-vitro* water release of MS (a) and MZ (b) formulations versus their technical products.

Figure 7 shows the cumulative release of MS and MZ from the clay-surfactant formulations as well as for the technical products. The release of herbicides presented an initial burst followed by a more gradual increase in the released amounts. This release pattern was strongly dependent on the herbicide, yielding larger released amounts for MZ in accord with its higher water solubility, compared to MS. Whereas the amount released from the technical product in the first five hours was 90% for MZ, the release for MS was 20% of the total amount. In addition, MS was not completely released during the course of the experiment, unlike MZ.

Clay-surfactant formulations showed a slower release relative to the technical products. The release of *a.i*. from clay-surfactant formulations can be modulated by the choice of the surfactant. The approach used can be extended to other clay-surfactant systems for encapsulation and slow release of target molecules of particular technological interest in agrochemical and pharmaceutical applications.

### Soil dissipation

The clay-surfactant formulation MSET/15 12/23/5 was chosen from the clay-surfactant formulation for bioassay tests because of its higher *a.i*. content and slower *in vitro* release. This formulation proved to be more bioactive in pot experiments relative to the commercial formulation (Table 6). This was due to the slower release of the active ingredient from the clay-surfactant formulation that prolonged MS bioactivity and improved weed control. At the field rate, the inhibition percent of the clay-based formulation was 68.9±2.2% amounting approximately to a 20% enhanced bioactivity relative to the commercial formulation, and as bioactive as this one when applied at two-fold the field rate (72.7±5.7%). At two-fold the field rate, the difference in the bioactivity of both formulations was reduced, but still higher for the clay-surfactant formulation.

**Table 6:** Inhibition percents (as measured by reduction in chlorophyll content relative to a control) of MS formulations applied at several rates in pot experiments.^a^

Rate applied	Commercial formulation	MSET/15 12/23/5
½-field rate	0	21.9±1.1
Field rate	55.3±2.2	68.9±2.2
2-field rate	72.7±5.8	83.0±1.6

The field rate was 100 g ha^−1^.

The commercial formulation was inactive at the lowest rate. On the contrary, the clay-surfactant formulation showed 21.9% inhibition. All these results indicate that lower doses may be applied from the clay-surfactant formulations to achieve a good weed control relative to the commercial formulation, with the subsequent reduction in environmental and economic costs.
